# Exploring the Overlap: Content Analysis of Hallucination and Delusion Scales

**DOI:** 10.1192/j.eurpsy.2025.500

**Published:** 2025-08-26

**Authors:** M. Goncharova, H. R. IJzerman, C. Jacquet, C. Dondé, C. Bortolon

**Affiliations:** 1 Psychology, University Grenoble Alpes, Grenoble; 2 Psychology, University Savoie Mont Blanc, Mont Blanc; 3 Institut Universitaire de France; 4 Psychiatry, University Grenoble Alpes; 5 INSERM; 6 Psychiatry, CHU Grenoble Alpes; 7 Psychiatry, CH Alpes-Isère; 8 Centre Référent Réhabilitation Psychosociale et Remédiation Cognitive, Grenoble, France

## Abstract

**Introduction:**

Psychosis is characterized by hallucinatory and delusional experiences. Although, it was mostly considered to be present among clinical populations, there is strong evidence it can also be found in the general population. Limited reviews currently exist on the quality of the assessment methods designed to evaluate psychotic-like experiences in the general population. None of them assessed whether the existing instruments measure the same construct and, consequently, neglected problems associated with the “jingle-jangle fallacy” (Weidman et al., 2017; Flake and Fried, 2020). This fallacy might account for contradictions in the literature, as well as, issues with generalizability of the results.

**Objectives:**

The goal of our study is to better understand the agreement between various instruments used to assess hallucinations and paranoia-like experiences.

**Methods:**

We conducted a systematic search of the scales assessing hallucinations and paranoia-like experiences among the general population. Labels for the content analysis were created based on their definition in literature by the first authors and revised by another researcher. Three researchers coded each item independently of each other. We then estimated to which extent any item overlaps with any item from the other scale included in the analysis. We used Jaccard index to assess similarities between sets (from 0 with no overlap among scales) to 1 (complete overlap). The analysis was done in R and Excel.

**Results:**

For 263 items from 11 hallucination scales, we estimated 38 labels with a mean overlap of 0.19 (very weak). CAPS demonstrated the highest mean overlap of 0.26. The highest overlaps were observed between MUSEQ and CAPS (0.5), MUSEQ and SPQ (0.4), and between LSHS-R and RHS (0.4). For the paranoia scales, the analysis of 183 items drawn from 12 scales resulted in 18 labels. The mean overlap across these labels was 0.30 - a weak association. The PIQ exhibited the highest mean overlap at 0.42, whereas the PSQ displayed the weakest overlap with a value of 0.17.

**Conclusions:**

The overlap between hallucination scales was very weak. This disparity may be due to different instruments adopt varying interpretations of the hallucination continuum (Laroi, 2012). The weak overlap in paranoia scales may be less problematic, as theoretical models and empirical data suggest a more clear continuum of suspiciousness within the general population (Freeman et al., 2005) that maps our results. It is necessary to establish certain common grounds regarding what experiences represent which sides of the continuum, both in their variability, and severity in the field.

**Disclosure of Interest:**

None Declared

